# Academic motivation and burnout among Chinese doctoral students: the mediating role of academic resilience and the moderating role of perceived stress

**DOI:** 10.3389/fpsyg.2026.1783669

**Published:** 2026-07-22

**Authors:** Geng Zhao

**Affiliations:** 1 Faculty of Education, Henan Normal University, Xinxiang, China; 2 Department of Public Order, Henan Police College, Zhengzhou, China

**Keywords:** academic burnout, academic motivation, academic resilience, doctoral students, perceived stress

## Abstract

**Background:**

Academic burnout is a persistent concern in doctoral education, but the associations among doctoral students' motivation, resilience, stress, and burnout may depend on how motivational resources operate under demanding conditions. Drawing on Self-Determination Theory and Conservation of Resources theory, this study examined whether academic resilience statistically mediated the association between academic motivation and academic burnout, and whether perceived stress moderated the motivation–resilience association.

**Methods:**

A cross-sectional online survey was completed by 525 currently enrolled doctoral students in China. Academic motivation was operationalized as a nine-item composite reflecting intrinsic interest, instrumental reasons for doctoral study, and clarity of purpose/low amotivation. Academic resilience, perceived stress, and academic burnout were assessed with context-adapted self-report measures. Regression models corresponding to PROCESS Models 4 and 7 were estimated with 5,000 bootstrap resamples while controlling for gender, training stage, discipline, and marital status.

**Results:**

Academic motivation was negatively associated with burnout (total effect *B* = −0.956, *p* < 0.001) and positively associated with resilience (*B* = 0.951, *p* < 0.001). Resilience was negatively associated with burnout after motivation was controlled (*B* = −0.446, *p* < 0.001), and the indirect association was significant [effect = −0.424, 95% bootstrap CI (−0.504, −0.344)]. The motivation × stress interaction was significant but opposite to the hypothesized attenuation pattern (*B* = 0.173, *p* < 0.001): the positive motivation–resilience association was stronger, not weaker, at higher perceived stress. The index of moderated mediation was −0.077, 95% bootstrap CI [−0.113, −0.043].

**Conclusion:**

Stronger academic motivation and resilience were associated with lower reported burnout, and the indirect association through resilience was statistically significant. The motivation–resilience association also varied across levels of perceived stress, although the observed direction differed from the original hypothesis. Given the cross-sectional design, adapted self-report measures, and very high intercorrelations among the focal variables, these findings should be regarded as preliminary evidence of conditional associations rather than as evidence of a distinct psychological process.

## 1 Introduction

Doctoral education requires sustained engagement with uncertain research problems, repeated evaluation, and long periods of self-directed work. These demands can be intellectually productive, but they also create conditions in which emotional exhaustion, detachment from research, and diminished academic efficacy may accumulate. Large studies and systematic reviews have documented substantial mental-health burdens among doctoral researchers and have linked those burdens to isolation, supervisory relationships, organizational conditions, and the prolonged uncertainty of doctoral work (Levecque et al., 2017; Evans et al., 2018; Hazell et al., 2020; Estupiñá et al., 2024). Burnout is therefore not only an individual wellbeing issue; it may also affect persistence, research engagement, and the sustainability of doctoral training systems (Litalien and Guay, 2015; Sverdlik et al., 2018; Cornér et al., 2021).

The Chinese doctoral context warrants specific attention. Doctoral candidates may face publication expectations, milestone requirements, career uncertainty, and hierarchical supervisory relationships, while supervisors themselves work within performance-oriented evaluation systems. Research on Chinese doctoral students has identified publication pressure, uncertain progress, financial concerns, and supervisory demands as salient sources of stress (Wang et al., 2019; Wang, 2022). These pressures do not affect all candidates in the same way. Some remain engaged and recover after setbacks, whereas others experience escalating exhaustion and detachment. Explaining this heterogeneity requires attention to both motivational engagement and the resources available for coping.

Motivation is central to doctoral persistence because research progress depends heavily on self-regulated effort. Self-Determination Theory (SDT) distinguishes intrinsic motivation, extrinsic regulation, and amotivation and emphasizes that the quality and internalization of motivation matter for sustained functioning (Deci and Ryan, 2000; Ryan and Deci, 2020). In practice, doctoral students often hold multiple motives simultaneously: curiosity and enjoyment may coexist with career goals, credential value, family expectations, and a desire for professional recognition. The present study therefore focuses on academic motivation as the overall strength and clarity of engagement in doctoral study, rather than treating motivation as purely intrinsic. The construct used here combines interest, perceived instrumental value, and low amotivation; it is not presented as a direct measure of autonomous motivation.

Academic resilience may help explain why motivation is associated with lower burnout. Resilience refers to the capacity to recover, persist, seek support, and adapt following academic difficulty. Doctoral research routinely involves rejected manuscripts, failed analyses, delayed data collection, and critical feedback. Motivated students may be more willing to interpret these events as problems to be managed rather than as reasons to disengage, while resilient responses may limit the accumulation of exhaustion and cynicism (Cassidy, 2016; Chamadia and Qureshi, 2021; Kokotsaki, 2023). This reasoning is consistent with Conservation of Resources (COR) theory, which proposes that resources tend to cluster and that access to one resource can support the maintenance or development of others (Hobfoll, 1989, 2011).

Perceived stress may further condition the motivation–resilience association. COR theory predicts that high demands can restrict resource investment because individuals must allocate attention and energy to immediate threats (Hobfoll et al., 2018). On that basis, the original theoretical expectation was that the positive association between motivation and resilience would weaken at high stress. However, an alternative pattern is also plausible: when pressure is high, differences in motivational engagement may become more consequential because strongly motivated students continue to mobilize coping resources while weakly motivated students disengage. Testing the interaction rather than assuming a universal motivational benefit is therefore important.

This study examines a moderated mediation model among Chinese doctoral students. It asks whether academic resilience statistically accounts for part of the association between academic motivation and burnout and whether perceived stress changes the strength of the motivation–resilience association. The study contributes by (a) examining motivation as a multidimensional engagement composite that reflects the mixed motives common in doctoral study, (b) testing resilience as an intervening correlate, and (c) evaluating whether the indirect association varies across perceived-stress levels. Because all variables were assessed at one time point, the model is interpreted as a conditional pattern of association rather than as evidence of temporal or causal mechanisms.

## 2 Theoretical framework and hypotheses

### 2.1 Academic motivation and academic burnout

SDT conceptualizes motivation along a continuum that ranges from autonomous interest to controlled regulation and amotivation (Deci and Ryan, 2000; Ryan and Deci, 2020). Although autonomous motives are generally more favorable for wellbeing, doctoral students commonly report blended reasons for continuing their studies. Curiosity, professional identity, career mobility, social recognition, and family expectations may all sustain effort. A broad academic-motivation score can therefore index whether students possess clear and active reasons for doctoral engagement, while acknowledging that it does not distinguish all forms of behavioral regulation.

Burnout reflects the erosion of energy and connection under prolonged demands, typically expressed through exhaustion, cynicism, and reduced efficacy (Maslach et al., 2001; Schaufeli et al., 2002). Students with stronger and clearer academic motivation may experience greater goal commitment, more persistent task initiation, and a stronger sense of meaning in research. By contrast, weak or uncertain motivation may make routine setbacks feel less tolerable and may increase psychological withdrawal. Evidence from higher education has associated stronger motivation with engagement and wellbeing, although effect sizes and temporal ordering vary across settings (Cook and Artino, 2016; Vansteenkiste et al., 2009; Trigueros et al., 2020).

H1: Academic motivation is negatively associated with academic burnout among Chinese doctoral students.

### 2.2 The mediating role of academic resilience

Academic resilience concerns adaptive responses to setbacks that threaten progress. It includes persistence, emotional recovery, problem solving, and appropriate help seeking. In doctoral education, resilience is not equivalent to enduring unlimited pressure; rather, it describes the capacity to respond flexibly while maintaining engagement with achievable academic goals (Cassidy, 2016; Kokotsaki, 2023).

From a COR perspective, motivation may function as an upstream personal resource that is associated with a greater willingness to invest effort in coping, seek feedback, and continue after failure. These responses are consistent with resilience. Resilience, in turn, may be associated with lower burnout because students who recover more effectively from setbacks are less likely to remain in prolonged states of exhaustion or detachment. The broaden-and-build perspective similarly suggests that interest and positive engagement can widen coping repertoires and support durable adaptive resources (Fredrickson, 2001). Because the present design is cross-sectional, mediation is used as a statistical decomposition of associations rather than as proof of a temporal process.

H2: Academic resilience statistically mediates the negative association between academic motivation and academic burnout.

### 2.3 Perceived stress as a moderator

Perceived stress reflects the extent to which demands are experienced as unpredictable, difficult to control, or greater than available coping capacity (Cohen et al., 1983; Lazarus and Folkman, 1984). In doctoral education, stress may arise from workload, publication demands, uncertainty about graduation, comparisons with peers, and concerns about employment. Stress is therefore not identical to objective workload; it represents an appraisal of the relation between demands and resources.

COR theory emphasizes the primacy of resource loss and proposes that resource investment becomes more difficult under strain (Hobfoll, 1989; Halbesleben et al., 2014). The original expectation was that high perceived stress would absorb cognitive and emotional capacity and weaken the positive association between motivation and resilience. Under lower stress, motivated students should have more room to translate engagement into adaptive coping. Under higher stress, immediate demands may compete with the effort required for reflection, help seeking, and recovery.

H3: Perceived stress moderates the positive association between academic motivation and academic resilience, such that the association is weaker at higher levels of perceived stress.

### 2.4 Moderated mediation

If perceived stress weakens the association between academic motivation and resilience, the indirect association between motivation and burnout through resilience should also become less negative as stress increases. This proposition can be evaluated through conditional indirect effects and the index of moderated mediation (Preacher et al., 2007; Hayes, 2018).

H4: The indirect association between academic motivation and academic burnout through academic resilience is weaker at higher levels of perceived stress.

## 3 Methods

### 3.1 Design, participants, and procedure

A cross-sectional online survey was administered through the Wenjuanxing platform on 26 November 2025. The survey link was distributed through WeChat channels used by doctoral students, including doctoral-student groups and peer forwarding. Institutional names were not collected, so the exact number of participating universities cannot be determined. Available Internet-protocol location records indicated participation from 19 provincial-level regions in China. The sample included students from research-intensive/Double First-Class universities, local universities, and research institutes involved in joint doctoral training.

Eligibility required participants to be currently enrolled doctoral students in China, able to read Chinese, and willing to provide electronic informed consent. The final training-stage category represented doctoral candidates who remained enrolled beyond the standard fourth year; no postdoctoral researchers were included. All 525 submitted questionnaires contained complete responses to the focal items and met the eligibility criteria. Because there was no defensible basis for excluding otherwise eligible cases, all 525 questionnaires were retained for analysis.

The sample included 308 men (58.7%) and 217 women (41.3%). There were 119 1st-year students (22.7%), 132 2nd-year students (25.1%), 112 3rd-year students (21.3%), and 162 students in the fourth year or beyond, including extended-status candidates (30.9%). Disciplines included science/engineering (22.3%), medicine (19.8%), education/psychology (25.3%), humanities (17.1%), and economics/management/law (15.4%). A total of 312 participants (59.4%) studied full-time and 213 (40.6%) were part-time or employed doctoral students. Most were unmarried (51.0%), while 37.1% were married without children and 11.8% were married with children. The study protocol underwent institutional administrative review before data collection; electronic informed consent and anonymity procedures are described in the Ethics statement.

### 3.2 Measures

All focal items used a five-point response scale (1 = strongly disagree/not at all true to 5 = strongly agree/completely true). The final Chinese items, English translations (Brislin, 1980), coding, and source rationale are provided in Supplementary Table 1. The measures were context-adapted for doctoral study. No separate pilot sample or formal expert-panel review was conducted before the main survey; this limitation is acknowledged below. No items were removed after examining the present data.

#### 3.2.1 Academic motivation

Academic motivation was assessed with nine items informed by the intrinsic motivation, extrinsic motivation, and amotivation domains of the Academic Motivation Scale (Vallerand et al., 1992). Three items assessed intrinsic interest (e.g., “Conducting academic research itself gives me pleasure and satisfaction”), three assessed instrumental or external reasons (e.g., “Obtaining a doctoral degree is very important for my future career development”), and three assessed clarity of purpose/low amotivation. Two negatively worded amotivation items were reverse-scored; a positively worded clarity item was scored in its original direction. The nine items were averaged so that higher scores represented stronger and clearer academic motivational engagement. This composite is not interpreted as a pure measure of autonomous or intrinsic motivation. Cronbach's alpha was 0.945. As a sensitivity analysis, the three-item intrinsic-motivation subscale was also analyzed separately (Supplementary Table 3).

#### 3.2.2 Academic resilience

Academic resilience was measured with eight doctoral-context items adapted from the CD-RISC framework and academic-resilience literature (Connor and Davidson, 2003; Yu and Zhang, 2007; Cassidy, 2016). Items assessed persistence, emotional recovery, help seeking, problem solving, and recovery following criticism or rejection. Two negatively worded items were reverse-scored. The item mean was used, with higher scores indicating greater reported resilience. Cronbach's alpha was 0.938.

#### 3.2.3 Perceived stress

Perceived stress was assessed with eight items adapted from the perceived-stress framework and Chinese PSS wording (Cohen et al., 1983; Lu et al., 2017). The items referred specifically to doctoral study, including workload, time pressure, graduation uncertainty, social comparison, research funding, and feeling overwhelmed. One positively worded coping item was reverse-scored. The mean of the eight items was used; higher scores indicated greater perceived academic stress. Cronbach's alpha was 0.939.

#### 3.2.4 Academic burnout

Academic burnout was assessed with nine items adapted from the MBI-Student Survey framework (Schaufeli et al., 2002; Hu and Schaufeli, 2009). Three items assessed emotional exhaustion, three assessed cynicism/detachment, and three assessed reduced academic efficacy. A positively worded engagement item and a positively worded efficacy item were reverse-scored. The overall item mean was used, with higher scores indicating greater burnout. Cronbach's alpha was 0.946.

#### 3.2.5 Covariates

Gender, doctoral training stage, discipline, and marital status were included as covariates to preserve comparability with the original model. Categorical variables were dummy-coded. 1st-year status, science/engineering, unmarried status, and male gender served as reference categories.

### 3.3 Data analysis

Data screening confirmed complete responses for the focal items. Means, standard deviations, Cronbach's alpha coefficients, skewness, kurtosis, and Pearson correlations were calculated. Because all focal variables were self-reported in the same questionnaire, common method variance was examined rather than assumed to be absent. An unrotated principal-components analysis was used as a descriptive diagnostic, and an item-level eight-factor confirmatory model (stress, three burnout facets, resilience, and three motivation facets) was compared with a single-factor model. The comparison was interpreted cautiously because global fit alone does not establish discriminant validity (Podsakoff et al., 2003).

The hypotheses were tested with ordinary least squares regression equations corresponding to Hayes's PROCESS Model 4 and Model 7 (Hayes, 2018). Continuous focal variables were mean-centered before interaction terms were calculated. The mediation model estimated the total association of motivation with burnout, the association of motivation with resilience, and the joint associations of motivation and resilience with burnout. The first-stage moderated mediation model estimated the motivation × perceived-stress interaction in the resilience equation. Conditional slopes and conditional indirect effects were calculated at the mean and at one standard deviation below and above the mean of perceived stress. Indirect effects and the index of moderated mediation were evaluated with 5,000 percentile bootstrap resamples. Two-tailed *p* values below 0.05 and confidence intervals excluding zero were considered statistically significant. Coefficients reported in the tables are unstandardized.

## 4 Results

### 4.1 Measurement diagnostics and common method considerations

Internal consistency was high for all four composite scores (Table 1). The item-level eight-factor model showed excellent global fit, χ^2^ (499) = 536.13, *p* = 0.121, CFI = 0.998, TLI = 0.998, RMSEA = 0.012. However, the single-factor model also showed excellent global fit, χ^2^ (527) = 564.02, *p* = 0.128, CFI = 0.998, TLI = 0.998, RMSEA = 0.012, and the chi-square difference was not significant, Δχ^2^ (28) = 27.89, *p* = 0.471. In addition, the first unrotated component accounted for 66.62% of item variance. These results do not support a claim that common method variance is negligible. Consequently, the following coefficients are interpreted as strong same-source associations, and the measurement overlap is treated as a central limitation rather than as a resolved diagnostic issue.

**Table 1:** Scale characteristics and reliability (*N* = 525).

Construct	Items	*M*	SD	Cronbach's α
Academic motivation	9	3.489	1.130	0.945
Academic resilience	8	3.491	1.149	0.938
Perceived stress	8	2.515	1.133	0.939
Academic burnout	9	2.535	1.143	0.946

All variables were scored from 1 to 5. Higher scores indicate stronger academic motivation, greater resilience, higher perceived stress, and greater burnout, respectively.

### 4.2 Descriptive statistics and correlations

Academic motivation and resilience were strongly positively correlated, while both were strongly negatively correlated with perceived stress and burnout. Perceived stress was strongly positively correlated with burnout (Table 2). All correlations were statistically significant at *p* < 0.001. The unusually large correlations reinforce the need for caution regarding shared-method variance and construct overlap.

**Table 2:** Means, standard deviations, reliability, and correlations.

Variable	*M*	SD	1	2	3	4
1. Academic motivation	3.489	1.130	(0.945)			
2. Academic resilience	3.491	1.149	0.940	(0.938)		
3. Perceived stress	2.515	1.133	−0.933	−0.938	(0.939)	
4. Academic burnout	2.535	1.143	−0.946	−0.942	0.938	(0.946)

*N* = 525. Cronbach's alpha coefficients appear on the diagonal in parentheses. All off-diagonal correlations are significant at *p* < 0.001.

### 4.3 Mediation analysis

Controlling for gender, training stage, discipline, and marital status, academic motivation was negatively associated with academic burnout (total association: *B* = −0.956, SE = 0.014, *p* < 0.001), supporting H1. Academic motivation was positively associated with academic resilience (*B* = 0.951, SE = 0.015, *p* < 0.001). When both motivation and resilience were included in the burnout equation, resilience was negatively associated with burnout (*B* = −0.446, SE = 0.037, *p* < 0.001), and the direct association of motivation with burnout remained significant (*B* = −0.532, SE = 0.038, *p* < 0.001).

The bootstrapped indirect association through resilience was −0.424 [BootSE = 0.041, 95% CI (−0.504, −0.344)]. The confidence interval excluded zero, supporting H2 as a statistical mediation hypothesis. The indirect component represented approximately 44.4% of the total association. Given the cross-sectional design, this result does not demonstrate that motivation temporally precedes resilience or that resilience causally reduces burnout. Complete mediation coefficients are reported in Table 3.

**Table 3:** Regression-based mediation analysis.

Equation/path	*B*	SE	*t*	*p*	95% CI	*R^2^*
Total: motivation → burnout (c)	−0.956	0.014	−66.080	< .001	[−0.984, −0.927]	0.897
Motivation → resilience (a)	0.951	0.015	62.518	< .001	[0.921, 0.981]	0.887
Resilience → burnout (b)	−0.446	0.037	−12.019	< .001	[−0.519, −0.373]	0.920
Direct: motivation → burnout (c')	−0.532	0.038	−14.162	< .001	[−0.605, −0.458]	0.920
Indirect effect (a × b)	−0.424	0.041	—	—	[−0.504, −0.344]	—

Unstandardized coefficients. All equations controlled for gender, training stage, discipline, and marital status. *R*^2^ for b and c′ refers to the same outcome equation. Indirect-effect SE and CI are bootstrap estimates based on 5,000 resamples.

### 4.4 Moderated mediation analysis

**Figure 1:**
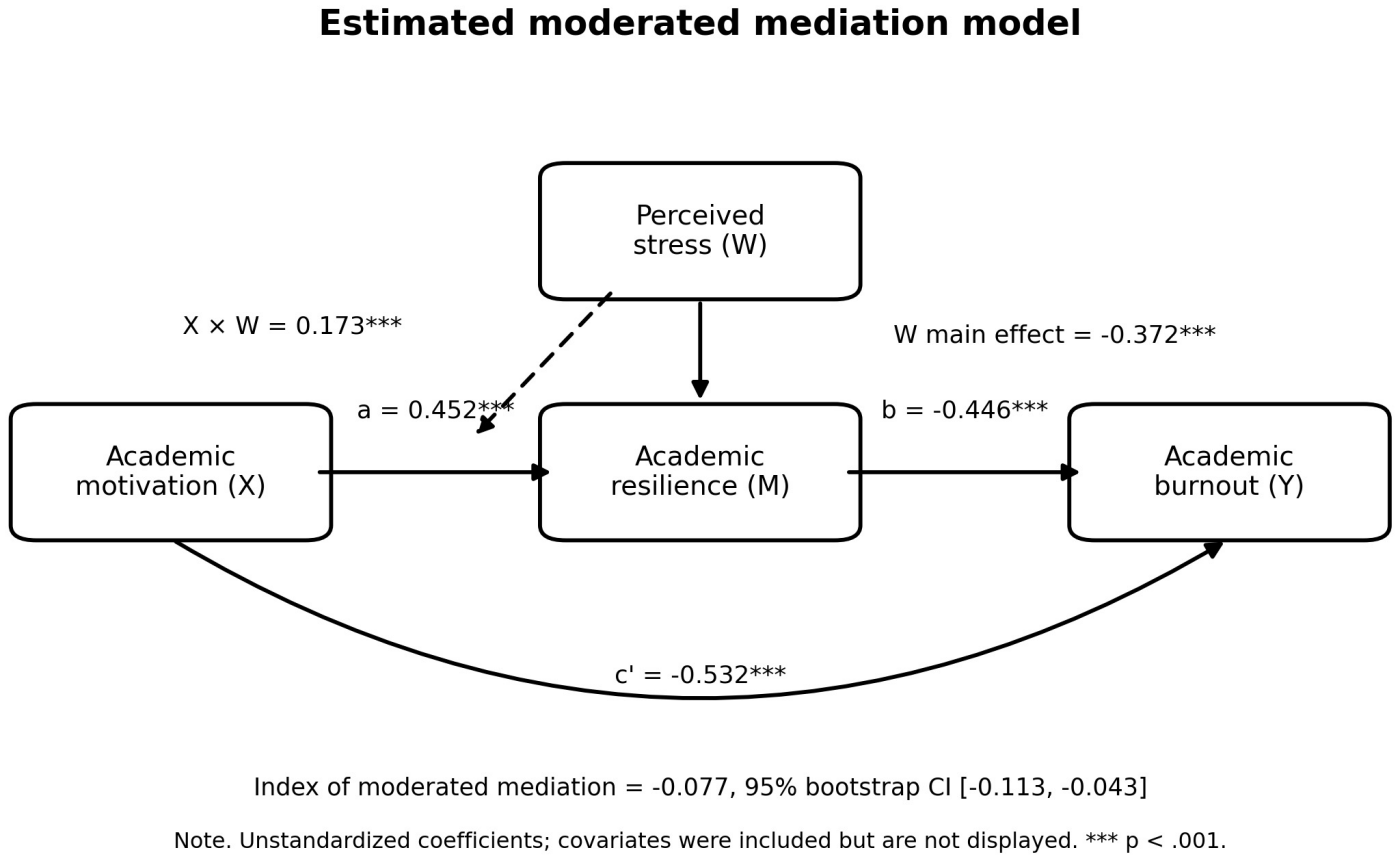
Estimated moderated mediation model linking academic motivation, academic resilience, perceived stress, and academic burnout. The figure presents the estimated first-stage moderated mediation model. Academic resilience was specified as the mediator of the association between academic motivation and academic burnout, and perceived stress was specified as the moderator of the association between academic motivation and academic resilience. Values shown are unstandardized regression coefficients. Gender, stage of doctoral study, discipline, and marital status were included as covariates but are not displayed for visual clarity. The index of moderated mediation and its 95% bootstrap confidence interval are reported below the model. ***p < 0.001.

**Figure 2:**
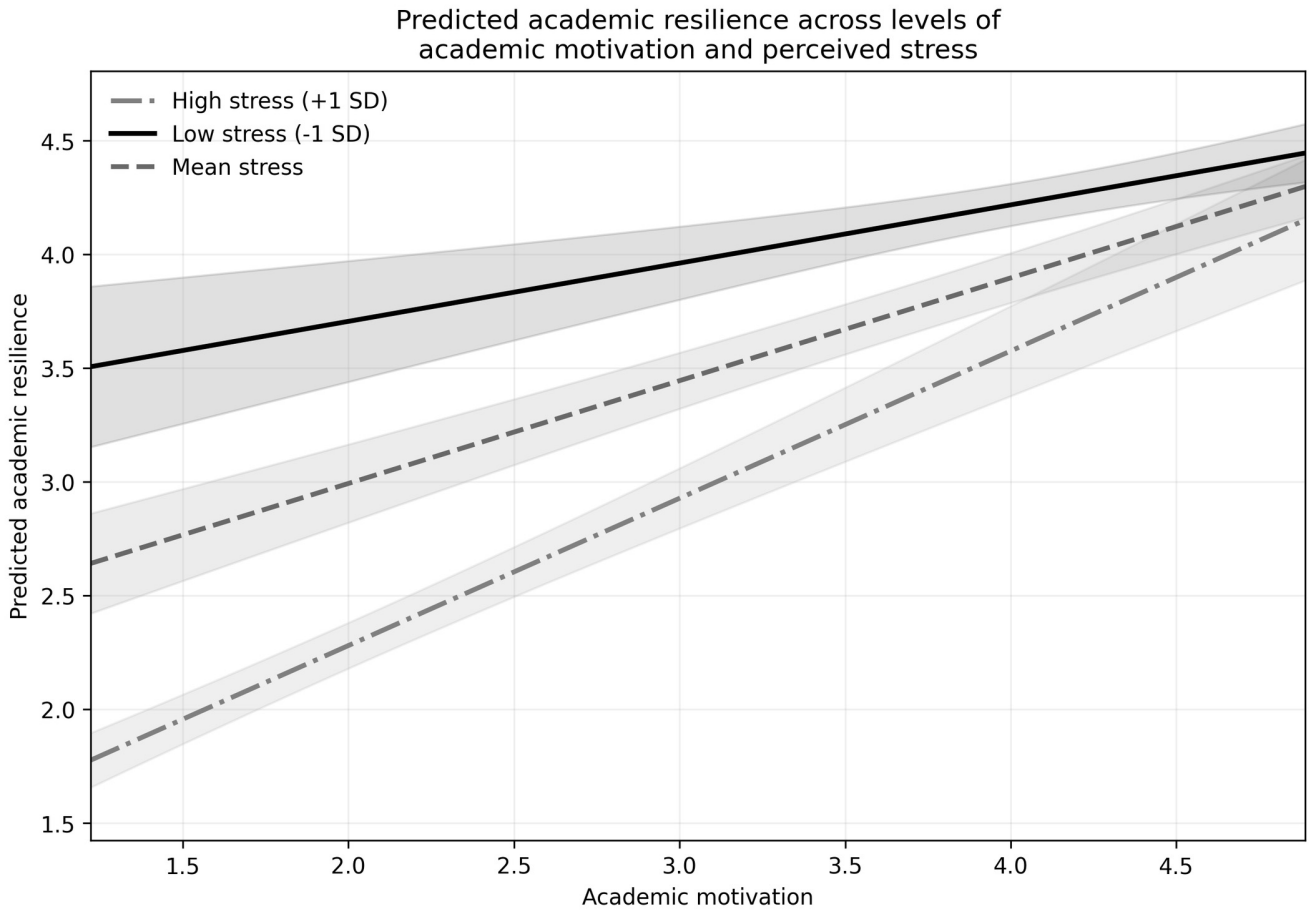
Interaction between academic motivation and perceived stress in predicting academic resilience. Predicted values of academic resilience are shown across the observed range of academic motivation at low perceived stress (−1 SD), mean perceived stress, and high perceived stress (+ 1 SD). Shaded bands represent 95% confidence intervals around the predicted values. The differing slopes indicate that the positive association between academic motivation and academic resilience varied across levels of perceived stress. Estimates were adjusted for gender, stage of doctoral study, discipline, and marital status.

The motivation × perceived-stress interaction significantly predicted academic resilience (B = 0.173, SE = 0.031, *p* < 0.001), increasing explained variance by Δ*R*^2^ = 0.0048, Δ*F*_(1, 511)_ = 30.16, *p* < 0.001. The interaction was positive, whereas H3 predicted attenuation at higher stress. H3 was therefore not supported in its hypothesized direction. The positive association between motivation and resilience was significant at low stress (*B* = 0.256, *p* < 0.001), mean stress (*B* = 0.452, *p* < 0.001), and high stress (*B* = 0.648, *p* < 0.001), and it became stronger as perceived stress increased. The moderation equation is reported in Table 4, and the estimated conditional-process model is presented in Figure 1. The simple slopes and corresponding confidence intervals are reported in Table 5 and visualized in Figure 2.

**Table 4:** First-stage moderation model predicting academic resilience.

Predictor	*B*	SE	*t*	*P*	95% CI
Academic motivation	0.452	0.038	11.884	< 0.001	[0.377, 0.527]
Perceived stress	−0.372	0.040	−9.307	< 0.001	[−0.450, −0.293]
Motivation × stress	0.173	0.031	5.492	< 0.001	[0.111, 0.235]

*N* = 525. Unstandardized coefficients. *R*^2^ = 0.918, adjusted *R*^2^ = 0.916, *F*(13, 511) = 442.76, *p* < 0.001. Covariates were gender, training stage, discipline, and marital status.

**Table 5:** Simple slopes of academic motivation predicting academic resilience.

Perceived stress level	*B*	SE	*t*	*p*	95% CI
Low (– 1 SD)	0.256	0.060	4.286	< 0.001	[0.139, 0.374]
Mean	0.452	0.038	11.884	< 0.001	[0.377, 0.527]
High (+ 1 SD)	0.648	0.043	15.008	< 0.001	[0.563, 0.732]

Low and high perceived stress correspond to one standard deviation below and above the sample mean. The high-stress slope is stronger rather than weaker.

The index of moderated mediation was −0.077 [BootSE = 0.018, 95% CI (−0.113, −0.043)], indicating that the indirect association varied across stress levels. The conditional indirect association was significant at low, mean, and high stress and became more negative as stress increased (Table 6). Thus, a significant conditional indirect pattern was observed, but H4 was not supported in its specified direction.

**Table 6:** Conditional indirect associations and index of moderated mediation.

Perceived stress level	Conditional indirect effect	BootSE	95% bootstrap CI
Low (−1 SD)	−0.114	0.032	[−0.180, −0.052]
Mean	−0.202	0.024	[−0.249, −0.157]
High (+ 1 SD)	−0.289	0.031	[−0.351, −0.229]
Index of moderated mediation	−0.077	0.018	[−0.113, −0.043]

Effects are unstandardized and based on 5,000 percentile bootstrap resamples.

## 5 Discussion

### 5.1 Summary of findings

This study examined how academic motivation, resilience, perceived stress, and burnout were associated among 525 Chinese doctoral students. Stronger academic motivation was associated with lower burnout and higher resilience, and resilience accounted statistically for part of the motivation–burnout association. These results supported H1 and H2 and were consistent with the view that clear reasons for doctoral engagement and adaptive responses to setbacks tend to co-occur with lower exhaustion, cynicism, and reduced efficacy.

The moderation findings did not support the original depletion-based prediction. Rather than weakening at higher stress, the positive association between motivation and resilience became stronger. The conditional indirect association through resilience also became more negative as stress increased. H3 and H4 were therefore not supported as formulated, even though the interaction and index of moderated mediation were statistically significant. This distinction is important: statistical significance of moderation does not confirm the hypothesized direction.

The observed pattern may be described as stress-contingent differentiation: the positive association between motivation and resilience was steeper at higher perceived stress. One possible interpretation is that motivational differences become more salient under demanding doctoral conditions. However, because the constructs were assessed concurrently using similarly formatted self-report items and were very highly correlated, the pattern may also reflect overlapping construct content or broader positive–negative response tendencies. The present data cannot distinguish between these explanations.

### 5.2 Theoretical implications

First, the results support treating doctoral motivation as multidimensional and practically mixed. Doctoral candidates may pursue research because it is interesting, because the degree has instrumental value, and because the goal is personally clear. The academic-motivation composite used here captures the strength and clarity of this engagement; it should not be interpreted as equivalent to intrinsic or autonomous motivation. This distinction narrows the theoretical claim relative to the original manuscript and aligns the construct label with the questionnaire actually administered.

Second, the indirect association is consistent with, but does not establish, the COR proposition that resources tend to cluster. Academic motivation and resilience were strongly related and were jointly associated with burnout. However, the magnitude of the intercorrelations raises uncertainty about whether they functioned as empirically distinct psychological resources in this dataset. Academic resilience should therefore be treated here as a statistical intervening variable rather than as a demonstrated psychological mechanism linking motivation to burnout.

Third, the positive interaction indicates that the association between academic motivation and academic resilience varied across levels of perceived stress. It should not, however, be interpreted as evidence that stress activates or strengthens a resource-investment process. The pattern may reflect a substantive conditional association, measurement overlap, or a combination of both. Replication using independently validated multidimensional measures, temporally separated assessments, and longitudinal designs is required before stronger theoretical conclusions are drawn.

### 5.3 Practical implications

Because the present study did not test interventions and the observed associations may partly reflect construct and method overlap, the following practical implications should be regarded as tentative directions for future evaluation rather than as effects demonstrated by the present data. Doctoral programs may consider supporting students in clarifying the personal, intellectual, and professional meaning of doctoral study. Structured goal review, career planning, and conversations about research identity may help students articulate reasons for continuing beyond external compliance.

Resilience support should not be reduced to exhortations to “be tougher.” Programs can provide methods consultation, writing groups, peer mentoring, and predictable feedback at proposal, mid-candidature, and pre-defense stages. Such supports may make adaptive coping and help seeking more feasible, particularly when students face repeated rejection or stalled progress. Autonomy-supportive supervision—clear expectations, meaningful rationales, and room for student input—may also help maintain engagement (Deci and Ryan, 2000; Panayidou and Priest, 2021).

Finally, the positive interaction does not imply that high stress is desirable. Perceived stress was strongly associated with burnout and negatively associated with resilience. Institutions should continue to examine avoidable stressors, including unclear milestone criteria, inconsistent supervisory practices, and rigid publication rules. The present findings only suggest that motivational differences may be especially visible under pressure; they do not show that increasing pressure improves resilience.

### 5.4 Limitations and future research

First, the cross-sectional design prevents temporal or causal inference. Motivation, resilience, stress, and burnout were measured simultaneously. Burnout may weaken motivation, resilience may shape stress appraisal, or reciprocal relationships may operate. Multi-wave designs, cross-lagged models, and intensive longitudinal methods are needed to establish temporal ordering.

Second, the study relied on a convenience sample recruited through WeChat on a single day. Institutional identifiers were not collected, the exact number of universities is unknown, and the sample may not represent the national doctoral population. Future studies should use stratified, multi-institution sampling and document recruitment flow prospectively.

Third, all constructs were assessed by self-report using similarly formatted items. The first component accounted for 66.62% of item variance, the single-factor model was not clearly inferior to the proposed item-level structure, and correlations among the composite scores were extremely high. Common method variance and limited discriminant validity are therefore substantial concerns. The large regression coefficients and R^2^ values should not be interpreted as precise estimates of distinct psychological processes. Future research should use validated full-length measures, temporally separated assessments, supervisor or peer reports, behavioral indicators, and objective progression data.

Fourth, the measures were shortened and adapted to the doctoral context without a separate pilot sample or formal expert-panel review. Although internal consistency was high and all items are disclosed, reliability alone does not establish construct validity. Future studies should conduct cognitive interviews, expert content review, exploratory and confirmatory validation in independent samples, and measurement-invariance testing across disciplines and training stages.

Fifth, the academic-motivation composite combines intrinsic interest, instrumental reasons, and low amotivation. It captures overall motivational engagement but cannot determine whether autonomous and controlled motives have different relations with resilience or burnout. Replication should use a full SDT-based instrument and test intrinsic motivation, identified regulation, introjected regulation, external regulation, and amotivation separately. The supplementary intrinsic-motivation sensitivity analysis showed the same positive interaction direction, but it does not resolve the measurement concerns.

Finally, the unexpected moderation pattern should be replicated before substantive conclusions are drawn. Future studies could preregister competing attenuation and amplification hypotheses, use prospective stress assessments around doctoral milestones, and test whether supervisory climate, funding security, or institutional evaluation practices explain when motivation is associated with resilient coping.

## 6 Conclusion

This study identified strong cross-sectional associations among academic motivation, academic resilience, perceived stress, and academic burnout in a sample of 525 Chinese doctoral students. Academic motivation was negatively associated with burnout and positively associated with resilience, while the indirect association through resilience was statistically significant. The association between motivation and resilience also varied across levels of perceived stress, although the observed direction was opposite to the original attenuation hypothesis.

These findings provide preliminary statistical evidence of an unexpected conditional pattern in doctoral students' self-reported academic experiences. They should not be interpreted as confirmation that motivation produces resilience, that resilience causally reduces burnout, or that perceived stress activates a distinct psychological resource process. Because the constructs were measured at a single point in time using adapted self-report instruments and showed very high intercorrelations, construct overlap and shared measurement variance remain plausible explanations for part of the observed pattern. The results therefore offer a basis for further investigation rather than a definitive account of the psychological mechanisms underlying doctoral burnout. Longitudinal, multi-source, and measurement-validation research is needed to determine whether these associations persist when motivation, resilience, stress, and burnout are more clearly distinguished.

## Data Availability

The raw data supporting the conclusions of this article will be made available by the authors, without undue reservation.
