# Supplementary material for: Academic motivation and burnout among Chinese doctoral students: the mediating role of academic resilience and the moderating role of perceived stress
Source: Front Psychol. 2026 Jul 22;17:1783669. doi: 10.3389/fpsyg.2026.1783669 (PMC13438418; doi:10.3389/fpsyg.2026.1783669)
Supplement: Supplementary file 1 [file Supplementary_file_1.docx]

# Supplementary Material

# Supplementary Table S1. Final questionnaire items, translations, and scoring

| **Construct** | **Code** | **Chinese item** | **English translation** | **Reverse** |
| --- | --- | --- | --- | --- |
| Perceived stress | Z1 | 我经常觉得博士阶段的学习和科研任务让我感到压力很大。 | I often feel that the learning and research tasks of doctoral study place me under substantial pressure. | No |
| Perceived stress | Z2 | 为了完成导师和课题组的要求，我常常感到时间严重不够用。 | I often feel that I have far too little time to meet the requirements of my supervisor and research group. | No |
| Perceived stress | Z3 | 我担心如果当前的科研进度持续下去，会影响我按时毕业。 | I worry that if my current research progress continues, it will affect my ability to graduate on time. | No |
| Perceived stress | Z4 | 与同领域的其他博士生相比，我经常担心自己“跟不上”。 | Compared with other doctoral students in my field, I often worry that I am falling behind. | No |
| Perceived stress | Z5 | 博士阶段的不确定性（如毕业、就业、晋升）经常让我感到紧张不安。 | Uncertainties in doctoral study, such as graduation, employment, and promotion, often make me anxious. | No |
| Perceived stress | Z6 | 我经常因为论文、项目或经费问题而感到心神不宁。 | I often feel distressed because of papers, projects, or funding problems. | No |
| Perceived stress | Z7 | 总体来说，我觉得自己能够比较从容地应对目前的学业压力。 | Overall, I feel that I can deal relatively calmly with my current academic pressure. | Yes |
| Perceived stress | Z8 | 我常常感到自己被各种学业与科研任务“压得喘不过气来”。 | I often feel overwhelmed by academic and research tasks. | No |
| Burnout—exhaustion | EE1 | 长期的博士学习和科研让我感到非常疲惫。 | Long-term doctoral study and research make me feel very tired. | No |
| Burnout—exhaustion | EE2 | 一想到要继续做当前的科研任务，我就感到精力被耗干。 | Thinking about continuing my current research tasks makes me feel drained. | No |
| Burnout—exhaustion | EE3 | 最近，我经常觉得自己已经“撑不住了”。 | Recently, I have often felt that I can no longer keep going. | No |
| Burnout—cynicism | CY1 | 有时我会对自己的研究课题感到冷漠或无所谓。 | Sometimes I feel indifferent toward my research topic. | No |
| Burnout—cynicism | CY2 | 我越来越常觉得做科研“只是完成任务”，而不是一件有意义的事情。 | I increasingly feel that doing research is only completing a task rather than doing something meaningful. | No |
| Burnout—cynicism | CY3 | 我仍然对自己的研究工作感到投入和兴趣。 | I remain engaged and interested in my research work. | Yes |
| Burnout—reduced efficacy | RE1 | 我经常怀疑自己是否有能力顺利完成博士学位。 | I often doubt whether I am capable of completing my doctoral degree successfully. | No |
| Burnout—reduced efficacy | RE2 | 即使我很努力，也很难在科研上取得让自己满意的成果。 | Even when I work hard, it is difficult to achieve research results that satisfy me. | No |
| Burnout—reduced efficacy | RE3 | 总体来说，我相信自己在博士阶段是有能力取得成功的。 | Overall, I believe that I am capable of succeeding during doctoral study. | Yes |
| Academic resilience | M1 | 即使在科研中多次遇到失败，我通常也能调整心态继续坚持。 | Even after repeated research failures, I can usually adjust and continue. | No |
| Academic resilience | M2 | 面对博士学习中的压力，我能比较快地从负面情绪中恢复过来。 | When facing pressure in doctoral study, I can recover relatively quickly from negative emotions. | No |
| Academic resilience | M3 | 遇到学业或科研困难时，我会主动寻求导师、同门或朋友的帮助。 | When I encounter academic or research difficulties, I actively seek help from my supervisor, peers, or friends. | No |
| Academic resilience | M4 | 一旦科研进展不顺利，我很容易就想放弃。 | When research progress is unsatisfactory, I easily feel like giving up. | Yes |
| Academic resilience | M5 | 我相信自己最终能够找到解决当前学术问题的办法。 | I believe that I will eventually find a way to solve my current academic problems. | No |
| Academic resilience | M6 | 压力大的时候，我通常能够找到一些办法让自己冷静下来。 | When pressure is high, I can usually find ways to calm myself. | No |
| Academic resilience | M7 | 之前经历的挫折让我在面对新困难时更加有韧性。 | Previous setbacks have made me more resilient when facing new difficulties. | No |
| Academic resilience | M8 | 遇到严厉的批评或论文被拒时，我很难从中恢复过来。 | I find it difficult to recover from harsh criticism or manuscript rejection. | Yes |
| Motivation—intrinsic | IM1 | 进行学术研究本身就让我感到愉悦和满足。 | Conducting academic research itself gives me pleasure and satisfaction. | No |
| Motivation—intrinsic | IM2 | 解决复杂的学术问题让我有很强的成就感。 | Solving complex academic problems gives me a strong sense of achievement. | No |
| Motivation—intrinsic | IM3 | 我读博主要是因为我真心对自己的研究领域感兴趣。 | I am pursuing a doctorate mainly because I am genuinely interested in my research field. | No |
| Motivation—instrumental | EM1 | 取得博士学位对我将来的职业发展（如升职、进高校）非常重要。 | Obtaining a doctoral degree is very important for my future career development. | No |
| Motivation—instrumental | EM2 | 家庭、导师或周围人的期望是我坚持读博的重要原因之一。 | The expectations of my family, supervisor, or people around me are an important reason for continuing doctoral study. | No |
| Motivation—instrumental | EM3 | 我希望通过博士学位获得更高的社会地位或认可。 | I hope to obtain greater social status or recognition through a doctoral degree. | No |
| Motivation—clarity | MC1 | 有时候我并不清楚自己为什么要继续读博。 | Sometimes I am not clear why I should continue doctoral study. | Yes |
| Motivation—clarity | MC2 | 我觉得自己读博“只是被动走到这一步”，而不是出于真正的选择。 | I feel that I arrived at doctoral study passively rather than through a genuine choice. | Yes |
| Motivation—clarity | MC3 | 我清楚地知道读博对我个人来说意味着什么。 | I clearly understand what doctoral study means to me personally. | No |

*Scoring: all items were rated from 1 to 5. Reverse-scored items were transformed as 6 - original response. Composite scores were calculated as item means. The academic-motivation composite included IM1-IM3, EM1-EM3, and MC1-MC3 after reverse scoring MC1-MC2.*

# Supplementary Table S2. Complete focal and covariate coefficients

| **Model** | **Predictor** | **B** | **SE** | **p** |
| --- | --- | --- | --- | --- |
| Total-effect burnout model | Academic motivation | -0.956 | 0.014 | < .001 |
|  | Female | 0.025 | 0.033 | .454 |
|  | Year 2 | -0.006 | 0.047 | .905 |
|  | Year 3 | -0.070 | 0.050 | .157 |
|  | Year 4+ | -0.009 | 0.046 | .850 |
|  | Medicine | -0.100 | 0.050 | .047 |
|  | Education/psychology | -0.021 | 0.047 | .654 |
|  | Humanities | -0.016 | 0.052 | .753 |
|  | Economics/management/law | 0.042 | 0.054 | .435 |
|  | Married, no children | -0.019 | 0.035 | .589 |
|  | Married, children | 0.058 | 0.053 | .267 |
| Mediator resilience model | Academic motivation | 0.951 | 0.015 | < .001 |
|  | Female | -0.015 | 0.035 | .668 |
|  | Year 2 | -0.013 | 0.050 | .791 |
|  | Year 3 | 0.077 | 0.052 | .141 |
|  | Year 4+ | 0.047 | 0.048 | .329 |
|  | Medicine | 0.117 | 0.053 | .026 |
|  | Education/psychology | -0.038 | 0.050 | .439 |
|  | Humanities | 0.062 | 0.055 | .259 |
|  | Economics/management/law | -0.038 | 0.057 | .508 |
|  | Married, no children | -0.017 | 0.037 | .641 |
|  | Married, children | -0.025 | 0.055 | .645 |
| Outcome burnout model | Academic motivation | -0.532 | 0.038 | < .001 |
|  | Academic resilience | -0.446 | 0.037 | < .001 |
|  | Female | 0.018 | 0.029 | .535 |
|  | Year 2 | -0.012 | 0.042 | .783 |
|  | Year 3 | -0.036 | 0.044 | .414 |
|  | Year 4+ | 0.012 | 0.040 | .761 |
|  | Medicine | -0.047 | 0.045 | .287 |
|  | Education/psychology | -0.038 | 0.042 | .359 |
|  | Humanities | 0.011 | 0.046 | .809 |
|  | Economics/management/law | 0.025 | 0.048 | .595 |
|  | Married, no children | -0.027 | 0.031 | .391 |
|  | Married, children | 0.047 | 0.046 | .312 |
| Moderation resilience model | Academic motivation | 0.452 | 0.038 | < .001 |
|  | Perceived stress | -0.372 | 0.040 | < .001 |
|  | Motivation × stress | 0.173 | 0.031 | < .001 |
|  | Female | 0.007 | 0.030 | .802 |
|  | Year 2 | -0.011 | 0.042 | .799 |
|  | Year 3 | 0.059 | 0.045 | .185 |
|  | Year 4+ | 0.018 | 0.041 | .653 |
|  | Medicine | 0.059 | 0.045 | .193 |
|  | Education/psychology | -0.038 | 0.042 | .372 |
|  | Humanities | 0.017 | 0.047 | .722 |
|  | Economics/management/law | -0.023 | 0.048 | .631 |
|  | Married, no children | -0.021 | 0.031 | .500 |
|  | Married, children | -0.009 | 0.047 | .856 |

*Reference groups: male, first-year doctoral student, science/engineering, and unmarried. Coefficients are unstandardized.*

# Supplementary Table S3. Sensitivity analysis using the three-item intrinsic-motivation subscale

| **Path/term** | **B** | **p** |
| --- | --- | --- |
| Intrinsic motivation → burnout (total) | -0.859 | < .001 |
| Intrinsic motivation → resilience | 0.852 | < .001 |
| Intrinsic motivation → burnout (direct) | -0.279 | < .001 |
| Resilience → burnout | -0.681 | < .001 |
| Intrinsic motivation × perceived stress | 0.125 | < .001 |

*The positive interaction direction remained when only the intrinsic-motivation items were used. This sensitivity analysis does not resolve common method or discriminant-validity concerns.*
